# Early Detection of Aphid Infestation and Insect-Plant Interaction Assessment in Wheat Using a Low-Cost Electronic Nose (E-Nose), Near-Infrared Spectroscopy and Machine Learning Modeling

**DOI:** 10.3390/s21175948

**Published:** 2021-09-04

**Authors:** Sigfredo Fuentes, Eden Tongson, Ranjith R. Unnithan, Claudia Gonzalez Viejo

**Affiliations:** 1 Digital Agriculture Food and Wine Group, School of Agriculture and Food, Faculty of Veterinary and Agricultural Sciences, University of Melbourne, Melbourne, VIC 3010, Australia; eden.tongson@unimelb.edu.au (E.T.); cgonzalez2@unimelb.edu.au (C.G.V.); 2 Department of Electrical and Electronic Engineering, School of Engineering, University of Melbourne, Melbourne, VIC 3010, Australia; r.ranjith@unimelb.edu.au

**Keywords:** remote sensing, volatile compounds, artificial neural networks, photosynthesis modeling, plant water status modeling

## Abstract

Advances in early insect detection have been reported using digital technologies through camera systems, sensor networks, and remote sensing coupled with machine learning (ML) modeling. However, up to date, there is no cost-effective system to monitor insect presence accurately and insect-plant interactions. This paper presents results on the implementation of near-infrared spectroscopy (NIR) and a low-cost electronic nose (e-nose) coupled with machine learning. Several artificial neural network (ANN) models were developed based on classification to detect the level of infestation and regression to predict insect numbers for both e-nose and NIR inputs, and plant physiological response based on e-nose to predict photosynthesis rate (A), transpiration (E) and stomatal conductance (gs). Results showed high accuracy for classification models ranging within 96.5–99.3% for NIR and between 94.2–99.2% using e-nose data as inputs. For regression models, high correlation coefficients were obtained for physiological parameters (gs, E and A) using e-nose data from all samples as inputs (R = 0.86) and R = 0.94 considering only control plants (no insect presence). Finally, R = 0.97 for NIR and R = 0.99 for e-nose data as inputs were obtained to predict number of insects. Performances for all models developed showed no signs of overfitting. In this paper, a field-based system using unmanned aerial vehicles with the e-nose as payload was proposed and described for deployment of ML models to aid growers in pest management practices.

## 1. Introduction

Early detection of insect infestation in crops is critical for decision-making related to pest management and alerting potential infestation to neighboring susceptible crops. One of the most common agronomical assessments for detrimental insect infestation in crops is visual at determined and critical periods of the crop development in synchronicity with the insect’s population dynamics [1] and migrations [2]. The next step for more practical monitoring is using pheromone traps [3], which can be used for more ecological pest management [4]. Some of these pheromone traps have been integrated with digital technologies, such as video cameras [5] to assess effectiveness [6] and implementing computer vision for pest identification and automatic counting using machine learning [7,8,9,10,11,12]. Some of these systems are web-based and used to support agronomical decision-making in developing countries [8].

Even though these applications are certainly an advancement in automated pest monitoring and management, they still rely on sentinel locations within the crop field. The latter could translate into an economic limitation for extensive crops, which require a significant number of monitoring nodes and increasing complexity of the sensor network. Furthermore, these monitoring and counting systems do not give much information on the insect-plant interaction, insect natural predator’s interaction, or detrimental effects or symptomatology from the plant’s perspective.

Other remote sensing techniques have been implemented for pest detection in crops [13] based on sensor networks [14], IoT for moths [15], hyperspectral imaging based on airborne [16], satellite [17], and unmanned aerial vehicles (UAV) [18], among others. These systems offer the advantage of increased spatial resolution and potential temporal resolution in airborne and UAV platforms. However, there are some disadvantages related to the plant-based nature of remote sensing monitoring. The first disadvantage is related to monitoring and modeling based on plant symptomatology in response to insect attacks, often assessed late, with detrimental implications in yield and quality of produce. Another disadvantage is that there is no assurance that symptomatology targeted using remote sensing to detect insects are entirely related to the specific biotic stress of interest. Some plants may have other biotic and abiotic symptomatology, such as water, salinity, and other insect interaction stresses. These issues could create biases in models developed and hinder capabilities of deployment of models to other locations.

Hence, there is a need for a digital system that considers the early detection of the pest of interest and early interaction with the host plant. To understand the specific insect-plant interactions for machine learning modeling purposes, controlled experiments must be considered before deployment in field conditions. Furthermore, a digital system based on volatile compounds could offer advantages compared to other systems. The implementation of electronic noses (e-noses) for insect detection have been proposed for disease detection and diagnosis [19] and pest detection [20], specifically for cotton [21], as a portable e-nose development, and specifically for aphid detection on tomato plants [22] using four low-cost gas sensors and comparing with gas chromatography results. In wheat, some authors have also used commercial e-noses to detect mite infestation [23] to predict the age and insect damageduring storage using linear discriminant analysis [24], and to detect rusty grain beetle, *Cryptolestes ferrugineus*, and red flour beetle in wheat [25]. Some studies have also combined computer vision systems and e-noses for pests in agriculture [26]. There is an increasing interest in developing compact, portable, and low-cost e-noses for these purposes [27]. However, most of these new researches are focused only on the detection of the variation in volatile compounds related to the insect presence and the interaction between insects and plants [25,28,29,30], and in some researches, combining e-nose and computer vision [26] for insect detection and identification, but so far, no attempt has been made to separate them through comprehensive modeling on these separate processes.

This paper proposed the implementation of a newly developed e-nose comprised of nine gas sensors described by Gonzalez Viejo et al. (2020) [31] and near-infrared spectroscopy (NIR) for the early detection of aphids (*Rhophalosiphum padi*) on wheat plants in controlled conditions. Raw data from the e-nose and NIR were used as inputs for machine learning algorithms to develop different classification models to detect insect’s presence at different phenological stages and regression models to predict the number of insects and physiological responses of plants based on gas-exchange measurements. Furthermore, a deployment system was proposed to validate these models in the field using the e-nose as a UAV payload to test different flying altitudes for detection sensitivity purposes. The latter system could have several advantages compared to research done so far by addressing the gaps discussed above.

The implementation of the proposed system can be highly beneficial to growers being able to provide high temporal and spatial resolution for more precise and targeted decision-making. Furthermore, the deployment of this system could support not only pest detection and management but also other agronomical activities, such as plant water status and irrigation scheduling and the detection of other biotic and abiotic stresses.

## 2. Materials and Methods

### 2.1. Plant and Insect Material, and Experimental Design Description

Wheat seeds of Kittyhawk variety (Pacific Seeds, Toowoomba, QLD, Australia) were surface sterilized with 0.8% sodium hypochlorite and were pre-germinated in the dark at 4 °C for 48 h, followed by lit conditions (17–25 °C) for 72 h. The germinated seeds were transferred individually to Jiffy-7^®^ pellets (Jiffy Products S.L. (Private) Ltd., Mirigama, Sri Lanka). The seedlings were further grown to a two-leaf stage (GS12) prior to transplanting in pots.

The plants were grown in a non-circulating passive hydroponic method based on Kratky [32]. The wheat seedlings were transplanted into 3 Li (190 mm × 170 mm) hydroponic pots (Anti-Spiral Pot, Garden City Plastics, Dandenong South, VIC, Australia) filled with expanded clay pebbles (CANNA Aqua Clay Pebbles, Subiaco, WA, Australia) as substrate, with three seedlings placed equidistant in each pot. Duplicate pots were placed in a black plastic tub filled with modified Hoagland nutrient solution [33] up to root submergence level. The nutrient solutions were replaced every two weeks throughout the experiment. Each tub of hydroponic set-up is placed inside an insect rearing tent (BugDorm, Australian Entomological Supplies Pty., Ltd., South Murwillumbah, NSW, Australia) constructed with nylon mesh with 160 µm aperture. The plants were maintained inside a growth room (Biosciences Glasshouse Complex, The University of Melbourne, Parkville, VIC, Australia) with 16 h daylight/8 h night and 20–25 °C controlled automatically.

Oat aphids (*Rhophalosiphum padi*) were obtained from laboratory cultures of Pest & Environmental Adaptation Research Group, School of Biosciences, The University of Melbourne, Australia. The starting colony was allowed to reproduce for population increase in a rearing tent supplied with wheat plants (in a similar hydroponic set-up described above). Adult *R. padi* were randomly selected from the colony plants and introduced into the experimental plants, approximately at stem elongation stage with third leaf emerged (GS32). Three treatments were determined based on the economic threshold for winter cereals which is an average of 15 aphids per tiller on 50% of tillers [34]: high load (15 aphids per tiller in 50% of tillers), medium load (10 aphids per tiller in 50% of tillers), and low aphid load (5 aphids per tiller in 50% of tillers). The aphids were carefully transferred into the wheat plants with a fine natural bristle brush. For simplicity, days referred in models developed correspond to days after infestation at the wheat phenological stage GS32.

**Figure 1:**
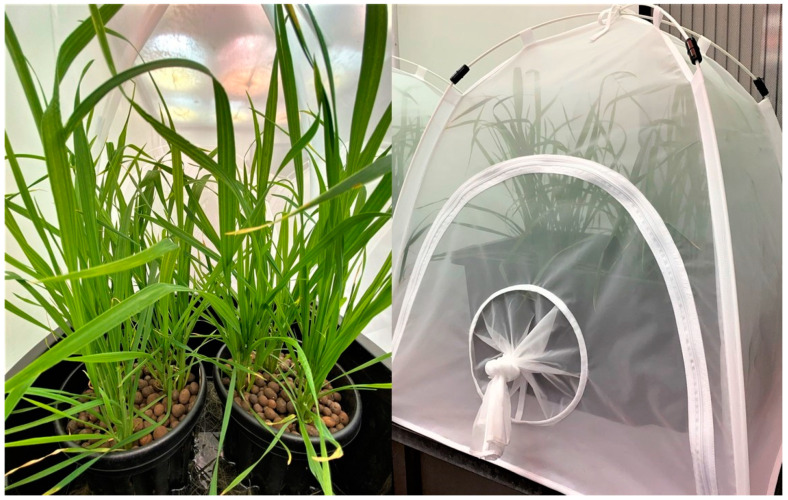
Wheat plants were grown in non-circulating passive hydroponic system (**left**) and contained in insect rearing tents (**right**).

A total of eight experimental set-ups were made with duplicate set-ups for each treatment (low, medium, and high aphid load) and two aphid-free set-ups as controls. Each experimental set-up was composed of one insect rearing tent, containing two pots with each pot planted with three wheat plants, maintained in hydroponics as described above and shown in Figure 1. These were randomly arranged inside the growth room.

Insect population models (adults) were developed using initial insect infestations and exponential growth models applicable to sigmoidal population insect growth [35,36]. Curves were adjusted by image analysis and manual insect counting per leaf, and extrapolation per plant in the middle and end of the experiment to account for insect mortality (data not shown).

### 2.2. Physiological Measurements

Plant physiological parameters such as stomatal conductance (gs; mol H_2_O m^−2^ s^−1^), transpiration (E; mmol H_2_O m^−2^ s^−1^), and photosynthesis (A; μmol CO_2_ m^−2^ s^−1^) were measured using a Li-6400 XT open gas exchange system (Li-Cor Inc., Environmental Sciences, Lincoln, NE, USA). Measurements were made on the youngest fully expanded leaves, repeated three times in different tillers of each plant (*n* = 18 per tent; *n* = 36 per treatment).

### 2.3. Near-Infrared Spectroscopy Measurements

**Figure 2:**
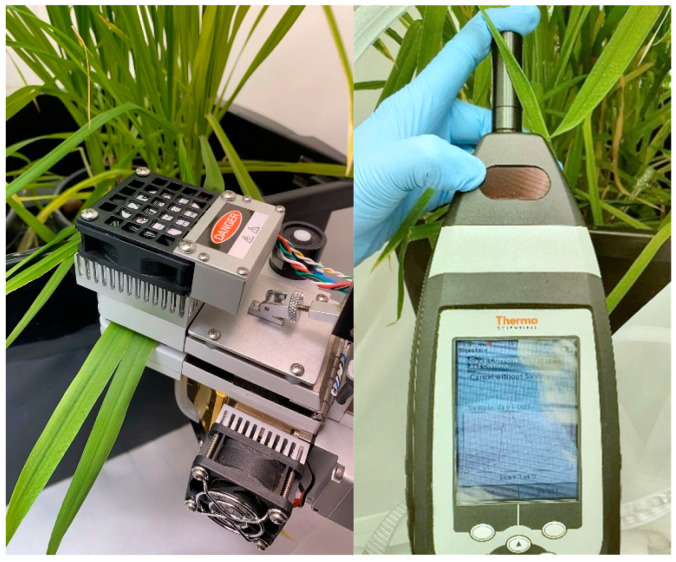
Photosynthetic gas exchange (**left**) and near-infrared (**right**) devices while taking measurements.

A single leaf of each wheat plant (three per pot and six leaves per tent) was measured on six different spots (*n* = 36 per tent; *n* = 72 per treatment) using a handheld near-infrared (NIR) spectroscopy device (MicroPHAZIR™ RX; Thermo Fisher Scientific, Waltham, MA, USA). This device measures the absorbance values within the 1596–2396 nm wavelength range. A blank reference was used as background to calibrate the device every 10 measurements and was placed on the top of the leaf while measuring to avoid recording noise from the environment (Figure 2). The raw absorbance values were used for all analyses presented in this study.

### 2.4. Electronic Nose Measurements

**Figure 3:**
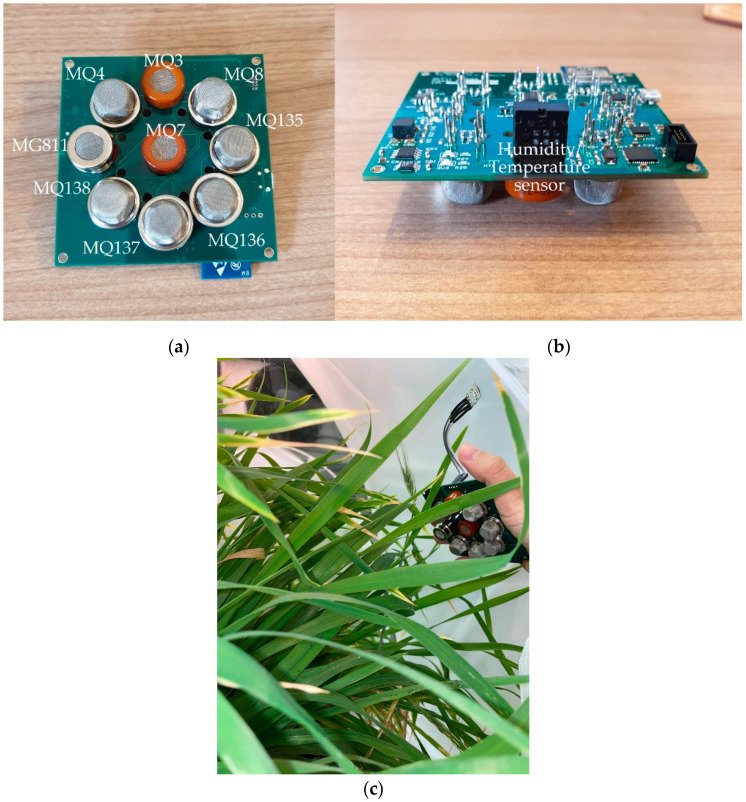
Electronic nose (e-nose) showing (**a**) the front part with gas sensors and their model ID (Henan Hanwei Electronics Co., Ltd., Henan, China) and (**b**) the back part which holds the humidity/temperature sensor; (**c**) Shows the e-nose positioning while taking measurements.

A portable and low-cost electronic nose (e-nose) developed by the Digital Agriculture Food and Wine Group and the Department of Electrical and Electronic Engineering from The University of Melbourne was used to assess volatile compounds produced by the control plants and treatments with aphids. This e-nose consists of an array of nine sensors sensitive to different gases: (i) MQ3 (alcohol), (ii) MQ4 (methane: CH_4_), (iii) MQ7 (carbon monoxide: CO), (iv) MQ8 (hydrogen: H_2_), (v) MQ135 (ammonia/alcohol/benzene), (vi) MQ136 (hydrogen sulfide: H_2_S), (vii) MQ137 (ammonia), (viii) MQ138 (benzene/alcohol/ammonia), and (ix) MG811 (carbon dioxide: CO_2_), as well as a humidity and temperature sensor to measure the environment conditions (Figure 3; Henan Hanwei Electronics Co., Ltd., Henan, China). The e-nose was calibrated for ~30 s prior to recording each measurement to ensure all sensors reached the baseline and then placed inside the tent on top of the plants to record data for 1.5 min; each tent was measured in triplicates. The output data (Volts) were then analyzed using a code written in MATLAB^®^ R2020a (Mathworks Inc., Natick, MA, USA) to extract the mean values of ten segments from the highest peak of the curves as described by Gonzalez Viejo et al. [37].

### 2.5. Statistical Analysis and Machine Learning Modeling

Physiological and e-nose data were analyzed using ANOVA to assess significant differences (*p* < 0.05) between samples; additionally, a Tukey honestly significant difference (HSD) *post hoc* test (α = 0.05) was conducted using XLSTAT v.2020.3.1 (Addinsoft, New York, NY, USA). These data were then analyzed for significant correlations (*p* < 0.05) based on covariance using MATLAB^®^ R2020a and represented with a matrix.

Several machine learning models based on artificial neural networks (ANN) were developed with three different purposes to (i) predict physiological data using e-nose outputs and the infestation level (control: 0, low: 0.25, medium: 0.75, and high: 1) as inputs using data from all treatments (Model 1), and only the baseline and control treatments (Model 2), (ii) classify samples into the different infestation treatments (control, low, medium, and high) using the NIR absorbance values (Models 3–7), and e-nose outputs (Models 8–12) as inputs, and (iii) predict the number of aphids using the NIR absorbance values (Model 13) and e-nose outputs (Model 14) as inputs. All models were constructed using a customized code written in MATLAB^®^ R2020a to test 17 different training algorithms in a loop and find the best models based on accuracy and performance [38,39]. Furthermore, a neuron trimming test (3, 5, 7, and 10 neurons) was performed to assess the most optimal number of neurons to avoid under- or over-fitting of the models (data not shown). The regression models (i, iii) consisted of a feedforward network with a hidden (tan-sigmoid function) and an output (linear transfer function) layer. On the other hand, the classification models (ii) consisted of a feedforward network with a hidden (tan-sigmoid function) and an output (Softmax neurons) layer.

**Figure 4:**
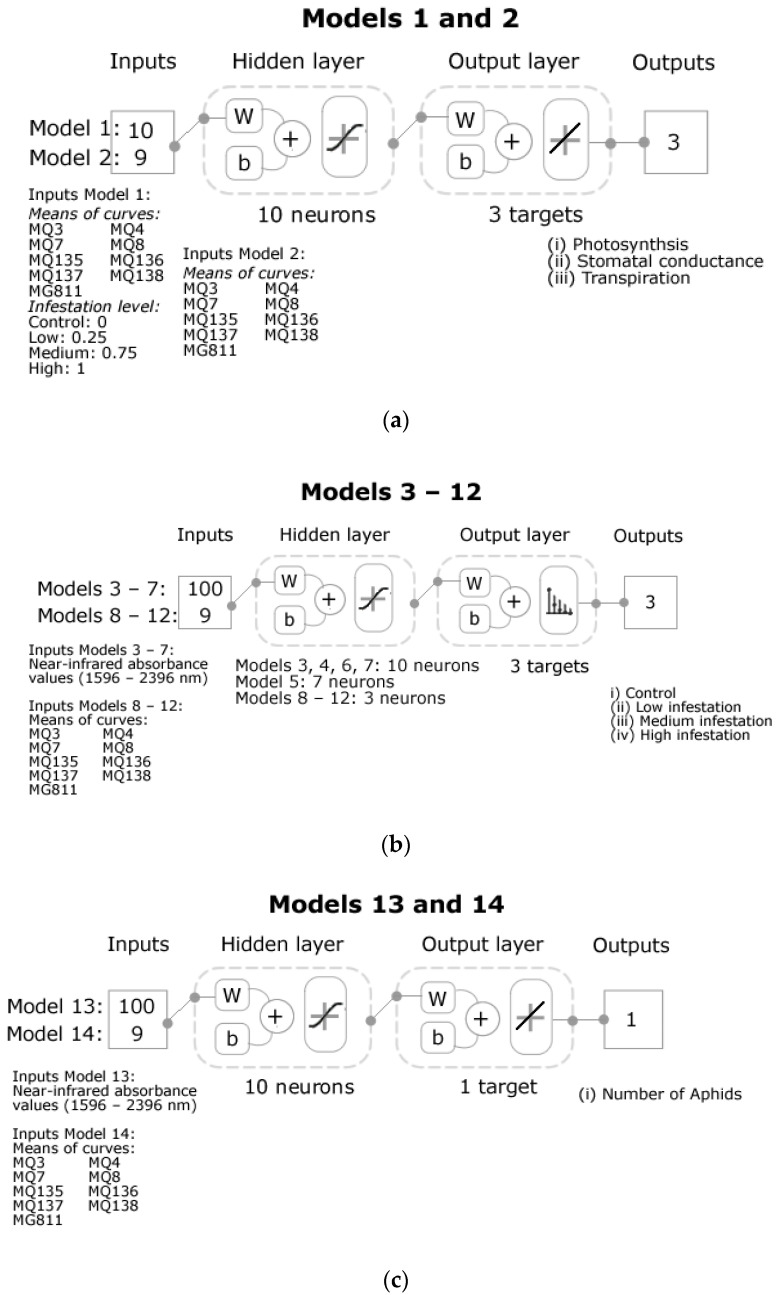
Diagrams of machine learning models based on artificial neural networks showing (**a**) the structure of regression Models 1 and 2; (**b**) Pattern recognition Models 3 to 12, and (**c**) Regression Models 13 and 14. Abbreviations: W: weights; b: bias; electronic nose sensors MQ3: alcohol; MQ4: methane; MQ7: carbon monoxide; MQ8: hydrogen; MQ135: ammonia/alcohol/benzene; MQ136: hydrogen sulfide; MQ137: ammonia; MQ138: benzene/alcohol/ammonia; MG811: carbon dioxide.

The best models to predict the physiological data (photosynthesis, stomatal conductance, transpiration) were developed using the Bayesian Regularization training algorithm for regression modeling. For this, two models were developed: Model 1 using as inputs the e-nose outputs and infestation level (control: 0, low: 0.25, medium: 0.75, and high: 1) from all measurements and treatments (general model), and Model 2 using the e-nose outputs from samples with no insects such as the baseline and control (Figure 4a). Data were divided randomly as 70% for training and 30% for testing using a performance algorithm based on means squared error (MSE).

Models to classify the samples into the different treatments (Figure 4b) using the NIR absorbance values as inputs were developed using the Levenberg–Marquardt training algorithm. One model was developed per day of measurement as Model 3 (baseline + Day 3), Model 4 (Day 7), Model 5 (Day 10), Model 6 (Day 14), and Model 7 (Day 17) to assess the level of infestation at different stages. Data were divided randomly as 70% for training, 15% for validation using a performance algorithm based on MSE, and 15% for testing. On the other hand, models to classify the samples into the different treatments using the e-nose outputs as inputs were constructed using the Bayesian Regularization training algorithm. Same as the previous, one model was developed per day of measurements as Model 8 (baseline + Day 3), Model 9 (Day 7), Model 10 (Day 10), Model 11 (Day 14), and Model 12 (Day 17). Data were also divided randomly as 70% for training and 30% for the testing stage using the MSE performance algorithm.

The Bayesian Regularization training algorithm produced the best models to predict the number of aphids using the NIR absorbance values (Model 13) and e-nose outputs (Model 14) from days 7 to 17 as inputs (Figure 4c). A random data division was used as 70% for training and 30% for testing with an MSE performance algorithm.

For Models 3–14, six support vector machine (SVM) algorithms (i) linear, (ii) quadratic, (iii) cubic, (iv) fine Gaussian, (v) medium Gaussian, and (vi) coarse Gaussian were also tested to compare results with ANN and find the best models. These algorithms were run using the Classification and Regression Learner applications in MATLAB^®^ Statistics and Machine Learning Toolbox 12.1. Accuracy percentage for classification and correlation coefficient (R) and MSE for regression models were considered to compare the different ML methods/algorithms. However, only accuracy percentage and R values are reported in results due to their lower accuracy compared to ANN. These algorithms were not tested for Models 1 and 2 because SVM algorithms are unable to construct multi-target models, which makes them inefficient for further deployment.

## 3. Results

Table 1 shows non-significant differences (*p* > 0.05) between treatments for baseline measurements of any physiological parameters. For photosynthesis, at days 10 and 17, the control was significantly higher (*p* < 0.05; 12.47 and 12.57 µmol m^−2^ s^−1^, respectively) than the infested treatments. Similarly, stomatal conductance was significantly higher for control at days 7, 10, and 17 (0.51, 0.55, and 0.62 mol m^−2^ s^−1^, respectively). On the other hand, transpiration was significantly higher for control in all measurement days (day 3–17) with values within the 3.60–6.00 mmol m^−2^ s^−1^ range.

**Figure 5:**
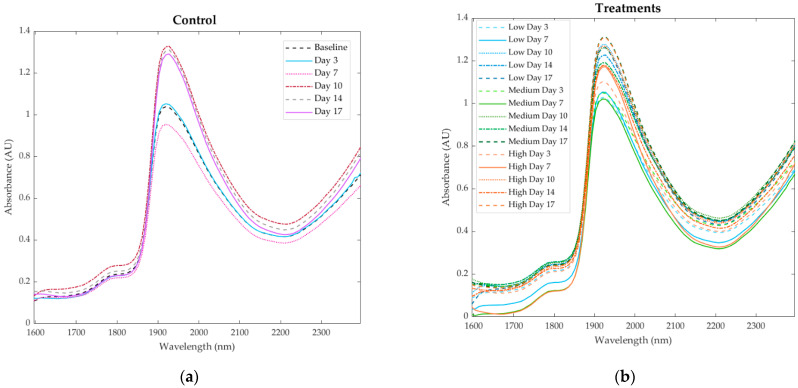
Near-infrared curves showing the absorbance values within the 1596–2396 nm wavelength range for (**a**) the control measurements and (**b**) the treatments (low, medium, high infestation) measured at different dates.

Figure 5a shows that the non-infested plants presented higher absorbance values at Days 10–17, especially within the 1900–2000 nm range, with Day 7 being the lowest. For the infested treatments (Figure 5b), the major overtones were also within the 1900–2000 nm range. The lowest absorbance values were at Day 7 for all low, medium, and high treatments, while the highest values were found at Day 17 for the medium and high treatments.

**Figure 6:**
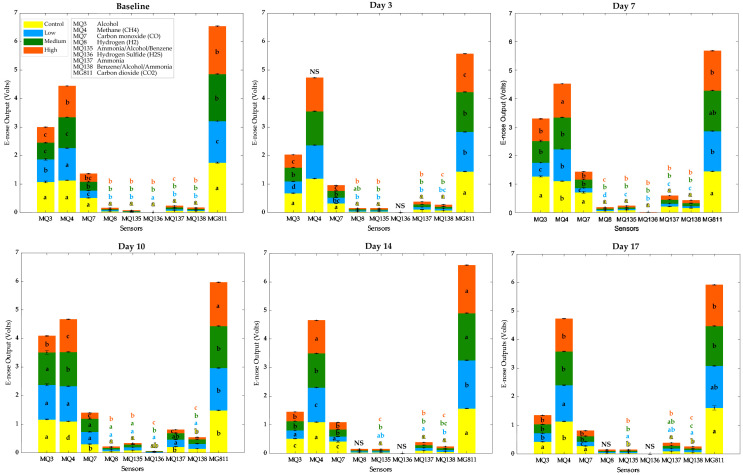
Electronic nose (e-nose) outputs (means) for each integrated sensor at each day of measurements. Error bars are based on standard error, and different letters denote significant differences between treatments according to ANOVA (*p* < 0.05) and Tukey honestly significant difference post hoc test (α = 0.05).

Figure 6 shows there were significant differences (*p* < 0.05) between treatments in all measurement days for all sensors, except for MQ4 (CH_4_) on Day 3, MQ136 (H_2_S) on Days 3, 14, and 17, and MQ8 (H_2_) at Days 14 and 17. It can be observed that the highest values were found in sensors MG811 (CO_2_), MQ4 (CH_4_), MQ3 (alcohol), and MQ7 (CO).

**Figure 7:**
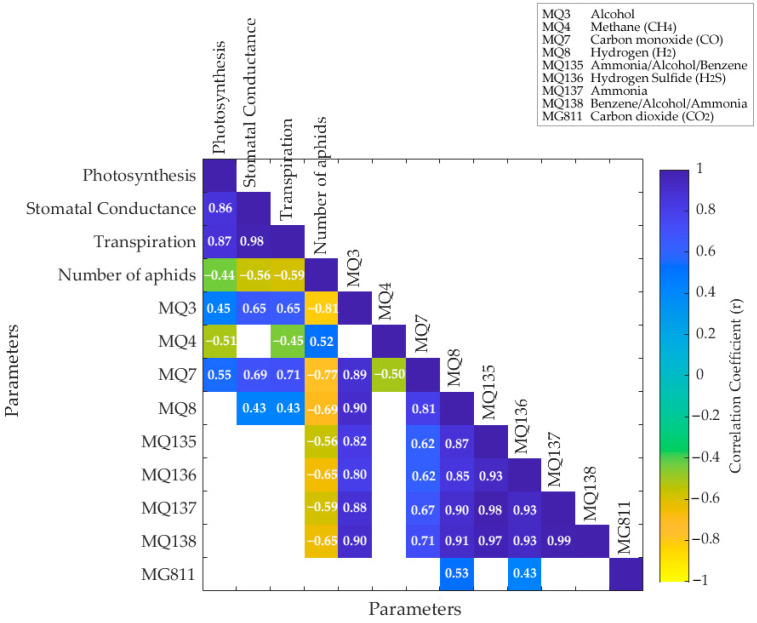
Matrix showing the significant correlations (*p* < 0.05) between the physiological data, number of aphids, and the electronic nose sensors. Color bar represents the negative (yellow) to positive (blue) correlations. Numbers within the boxes denote the correlation coefficients (r).

Figure 7 shows that both photosynthesis and transpiration had a positive and significant correlation (*p* < 0.05) with MQ3 (alcohol; r = 0.45 and r = 0.65, respectively), and MQ7 (CO; r = 0.55 and r = 0.71, respectively), and a negative correlation with number of aphids (r = −0.44 and r = −0.59, respectively) and MQ4 (CH_4_; r = −0.51 and r = −0.45, respectively). Similarly, stomatal conductance had a positive correlation with MQ3 (r = 0.65) and MQ7 (r = 0.69), and a negative correlation with number of aphids (r = −0.56). Transpiration and stomatal conductance were also positively correlated with MQ8 (H_2_; r = 0.43). On the other hand, number of aphids had a positive correlation with MQ4 (r = 0.52) and a negative correlation with MQ3, MQ7, MQ8, MQ135, MQ136, MQ137, and MQ138 with correlations within the r = −0.56–−0.81 range.

**Figure 8:**
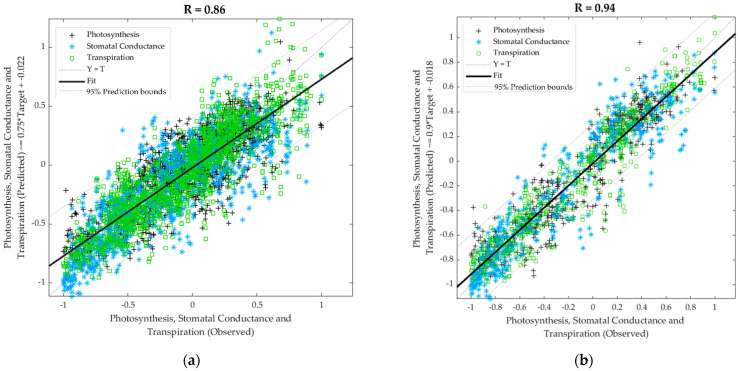
Overall regression models to predict physiological data using (**a**) the electronic nose outputs and infestation level as inputs for general data using all treatments at all measurement days and (**b**) using the electronic nose outputs as inputs with the baseline and control data (non-infested). Abbreviations: R: correlation coefficient; T: targets.

Table 2 shows the results from the machine learning regression models to predict physiological data (photosynthesis, stomatal conductance, and transpiration) using the e-nose outputs and infestation level as inputs. Model 1 was constructed as a general model using data from all treatments, and measurement days had an overall correlation coefficient R = 0.86. It had no signs of under- or overfitting as the MSE value of the training stage (MSE = 0.05) was lower than the testing (MSE = 0.06); however, the slope values were medium (b = 0.76). On the other hand, Model 2, which was developed using only the data from non-infested plants (baseline and controls), had high overall accuracy (R = 0.94) with high slope values (b = 0.90) and no signs of under- or overfitting with training MSE = 0.02 lower than testing MSE = 0.04. The overall models are shown in Figure 8, where data points from Model 1 (Figure 8a) are more dispersed and had 5% of outliers (216 out of 4320) based on the 95% prediction bounds. Model 2 (Figure 8b) also presented 5% of outliers (81 out of 1620), but the slope was closer to the unity (b = 0.90). It can also be observed that for Model 2, most of the outliers were from stomatal conductance, while in Model 1, they were more similar for the three targets.

Table 3 shows the results from the pattern recognition models to classify samples into the different treatments (control, low, medium, and high) using the NIR absorbance values as inputs. It can be observed that Model 3 was constructed using data from the baseline and Day 3, and Model 6 was developed with data from Day 14; both had a very high overall accuracy of 97%, being the lowest in accuracy compared to the other days of measurement. Model 4 was developed using data from Day 7 presented a higher overall accuracy of 98%. On the other hand, Models 5 and 7 had the highest overall accuracy (99%), with Model 5 being the best as it was constructed using a lower number of neurons (Model 5: 7 neurons; Model 7: 10 neurons). None of the five models presented any signs of under- or overfitting, and the MSE values of training (MSE < 0.01 for all) were lower than the validation and testing, and the latter stages had similar MSE values.

Accuracy results for Models 3–7 using SVM were lower than those from ANN Levenberg–Marquardt algorithm (Table 3). Results were within the following ranges: (i) Linear SVM (Models 3–7: 56–74%), (ii) Quadratic SVM (Models 3–7: 80–95%), (iii) Cubic SVM (Models 3–7: 90–92%, 88–98%), (iv) Fine Gaussian SVM (Models 3–7: 82–83%), (v) Medium Gaussian SVM (Models 3–7: 58–65%), and (vi) Coarse Gaussian SVM (Models 3–7: 41–45%). As can be seen, the model with the highest accuracy was with quadratic SVM (98%). However, this is lower than the ANN models, which presented the highest accuracy of 99.3%.

Table 4 shows the results from the pattern recognition models to classify samples into the different treatments (control, low, medium, and high) using the e-nose outputs as inputs. It can be observed that Models 8, 9, and 11 were developed using data from days 3, 7, and 14, respectively, and had very high overall accuracy (98%). Whilst Model 10 constructed with data from Day 10 presented the highest overall accuracy (99%). On the other hand, Model 12, developed using data from the last day of measurements (Day 17), presented high overall accuracy of 94%; however, it was the lowest compared to models from previous days. All of the models were constructed using three neurons, and none of them presented signs of under- or overfitting as the MSE values of the training stage (MSE < 0.01) were lower than the testing.

Accuracy results for Models 8–12 using SVM were lower than those from ANN Bayesian Regularization algorithm (Table 4). Results were within the following ranges: (i) Linear SVM (Models 8–12: 75–85%), (ii) Quadratic SVM (Models 8–12: 84–96%), (iii) Cubic SVM (Models 8–12: 88–98%), (iv) Fine Gaussian SVM (Models 8–12: 89–93%), (v) Medium Gaussian SVM (Models 8–12: 85–94%), and (vi) Coarse Gaussian SVM (Models 8–12: 72–85%). As can be observed, the model with the highest accuracy was cubic SVM (98%). However, this was lower than the ANN models, which presented the highest accuracy of 99.2%.

**Figure 9:**
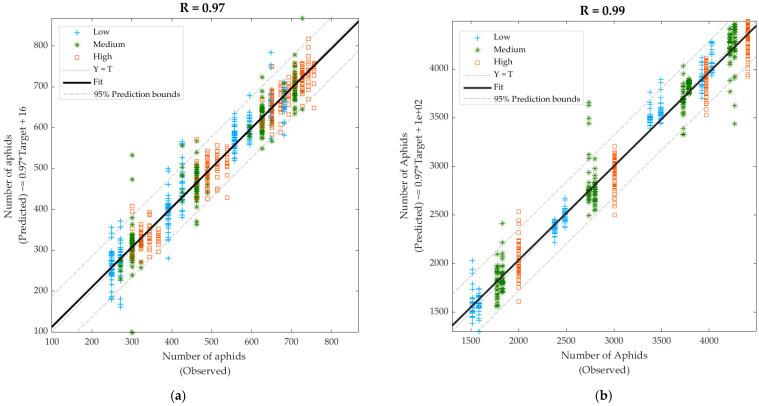
Overall regression models to predict the number of aphids using (**a**) the near-infrared absorbance values and (**b**) the electronic nose outputs as inputs with data from Days 7–17. Abbreviations: R: correlation coefficient; T: targets.

Table 5 shows the results from regression models to predict the number of aphids using data from Days 7 to 17. It can be observed that Model 13, developed using NIR absorbance values as inputs, had a very high overall correlation coefficient (R = 0.97). However, Model 14, constructed with the e-nose outputs as inputs, presented higher accuracy (R = 0.99). Both models had very high overall slope values (b = 0.97), and none showed any signs of under- or overfitting based on the performance values. From the overall models, Model 13 (Figure 9a) had 4.98% of outliers (43 out of 864) based on the 95% prediction bounds with the highest number of outliers due to the low infestation treatment. Similarly, Model 14 (Figure 9b) presented 5% of outliers (36 out of 720); however, for this model, the highest number of outliers was due to the medium infestation treatment. The difference in the number of aphids (target) between both models relies on the samples/measurements as NIR was measured on each plant, while e-nose was measured per tent.

Correlation coefficients for Models 13 and 14 using SVM were lower than those from ANN Bayesian Regularization algorithm (Table 5). Results from regression SVM were the following: (i) Linear SVM (Model 13: R = 0.68; Model 14: R = 0.80), (ii) Quadratic SVM (Model 13: R = 0.85; Model 14: R = 0.91), (iii) Cubic SVM (Model 13: R = 0.95; Model 14: R = 0.89), (iv) Fine Gaussian SVM (Model 13: R = 0.80; Model 14: R = 0.97), (v) Medium Gaussian SVM (Model 13: R = 0.73; Model 14: R = 0.92), and (vi) Coarse Gaussian SVM (Model 13: R = 0.60; Model 14: R = 0.70). It can be observed that for the model developed using NIR inputs, i.e., Model 13, the highest accuracy was with cubic SVM (R = 0.95), while for the model developed using e-nose inputs, i.e., Model 14, the highest accuracy was obtained with medium Gaussian SVM (R = 0.97). However, these were presented with lower accuracies than the ANN models which presented R values of 0.97 (Model 13) and 0.99 (Model 14).

## 4. Discussion

### 4.1. Physiological Response of Plants to Insect Infestation

Having no statistical differences in physiological data for the baseline with no insects for all plants (Table 1) helped ascertain that those initial conditions were similar for all the plants considered in the study, and no other stresses were present. Differences in physiological data after the introduction of insects in different treatments followed a variable pattern with not much difference for the photosynthetic rate (A), which is expected since plants compensate by either maintaining or increasing in some conditions A due to abiotic [40,41] or biotic stresses such as aphid attack [42].

In the case of stomatal conductance (gs) and transpiration, there were decreasing values according to the level of insect infestation, which is in accordance with previous studies, which have shown that gs is the most sensitive parameter to other stresses, such as water stress [43,44], pathogen-based stress [45], and water stress–aphid interactions in wheat [46].

### 4.2. Chemical Fingerprinting and Volatile Compounds’ Response to Insect Infestation

The NIR measurements offer a chemical fingerprint of the different leaf samples monitored, including the baseline measurements (Figure 5a) and treatments (Figure 5b) for the different days of the experimental trial. The main variations observable are in the overtones corresponding to hydrogen peroxide (H_2_O_2_) in the range of 1596 and 1650 nm [47,48] with similar absorbance levels for all treatments, which may explain the lower effect on photosynthesis reductions. The overtones for water content (status) are shown in the major peak within 1900–2000 nm (1940 nm) [49]. Furthermore, overtones of aromatic compounds can be found in the range of the NIR instrument sensitivity, at 1660 nm, 1672 nm, and 1685 nm [50,51]. Compounds with amide functional groups are at 1920 nm, 1960–1980 nm, 2000–2050 nm, and 2110–2160 nm [51,52]. Overtones of urea, which is an important amide compound, are found at 1990 nm, 2030 nm, and 2070 nm [51]; this was expected to be found in the samples as it is a nitrogen component contained in fertilizers added to the hydroponic solution, which is translocated through the plants.

In the case of e-nose (Figure 6), the baseline data were similar for plants and all tents measured. However, some differences between tents were statistically significant, contrary to the physiological data measured by gas exchange (Table 1). This can be explained by the sensitivity and responsiveness of e-nose sensors (every 0.5 s), which depend on small eddies formed in the growth chamber. Some sensors were more stable than others, such as MQ4 for Day 3, corresponding to methane sensitivity. The differences in sensor readings for subsequent days are expected, and it is assumed that their patterns are related to the interaction between aphids and plants and the increased number of insects in time and plant growth/decline, even small changes in the MG811 (CO_2_), which corresponds to photosynthetic activity.

When analyzing the correlations between physiological parameters and the sensors that compose the e-nose (Figure 7), it can be seen that, as expected, there is a positive and direct correlation between photosynthesis, stomatal conductance, and transpiration. On the contrary, there is an inverse correlation between physiological parameters and the number of insects, which corresponds to the decline of the plants or response to insect activity. Alcohol has been documented to be produced in plants as an allelopathic response to insect infestation. However, salivary proteins from aphids are able to stop this process when feeding on the plant sap [53,54]. The latter effect may explain the inverse correlation between the number of insects and the MQ3 sensor response signal. Furthermore, methane (MQ4) signal response increase may be due to activity of insects and anaerobic digestion [55], which explains the inverse correlations with physiological parameters and positive correlations with the number of insects. The carbon monoxide sensor (MQ7) had a similar response as the alcohol sensor (MQ3), shown by the high correlation coefficient (r = 0.89) and MQ8 (hydrogen). Most of the other sensors (MQ135, MQ136, MQ137, and MQ138) had an inverse correlation with the number of insects. The inverse correlation between the ammonia sensors (MQ135, MQ137, and MQ138) may be due to the capacity of aphids to assimilate ammonia into amino acids [56]. Finally, the levels of CO_2_ (MG811) were not significantly affected by the interaction between insects and plants. The correlations among the different sensors from the e-nose have been reported in previous research [31], which explains in detail the e-nose used in this study.

### 4.3. Machine Learning Models Developed

The plant physiological machine learning model developed from e-nose data as inputs and LiCOR data as targets for all plants and treatments (Figure 8a), and only control plants (Figure 8b) showed high correlation coefficients and no signs of overfitting. As far as authors ’ knowledge, this is the first time these models are presented, which use a low-cost e-nose compared to an established gas exchange method for plant physiological measurements. The LiCOR instrument has been used as a validation method for several remote sensing techniques for other crops [57,58,59,60]. The accuracy of the models obtained may not be surprising since both systems, LiCOR and the e-nose, measure gas exchange in different ways. Furthermore, these models are supported by the correlations between different sensors and physiological parameters (Figure 7). The lower correlation found in the ML model, including all plants (R = 0.86), may be explained by the higher variability of the data due to the interaction between plant and insect. Both models may be used to assess the level of the effect of plant-insect interaction on physiological parameters and for further applications to assess plant water stress [61], irrigation scheduling, and the physiological effect of other biotic or abiotic stresses, such as salinity, other insects, plant diseases, and environmental stress such as heatwaves, cold temperatures, and smoke contamination due to bushfires [62].

The accuracy of classification ML models based on NIR and e-nose data as inputs and level of insect infestation as targets was high and similar with over 94% accuracy for all models and dates, with slightly higher accuracies for ML models based on e-nose (Table 3 and Table 4, respectively). Within the most important are Models 3 and 8, respectively, since they can be considered for early detection only after three days of insect introduction to the plants’ environments and the corresponding treatments in a critical and vulnerable wheat phenological stage. In these models specifically, the baseline data from all plants were used as control, which explained the higher number of samples (576 and 480, respectively). Even though there was unbalanced data for the treatments as classifiers, the models were able to recognize non-infected plants with 96.5% and 98.3%, respectively. All further models can be used either to monitor insect activity or to verify the effectiveness of control methods using either chemical pesticides [63], organic pesticides [64,65], and natural predators through integrated pest management (IPM) [66,67].

### 4.4. Deployment Method for ML Models Developed Proposed Using UAV

One of the main advantages of creating AI models for the early detection of pests using growth chambers is that data can be obtained in control conditions. Hence the ML models developed do not include stresses related to other biotic or abiotic factors. The similarity of models developed using NIR and e-nose validate the effectiveness of the low-cost instrumentation proposed by comparing them with more established instruments, such as NIR spectroscopy; other studies have used, as a validation point, gas chromatography [20,22].

One advantage of the NIR models, especially for insect number detection, is that they are based on the different patterns of chemical fingerprinting resulting from the plant-insect interactions. Hence, this instrument can be used as a validation method to deploy the ML models developed in this study to field conditions. NIR measurements in plant leaves take just seconds and can be made on a grid of 10 × 10 m in a wheat field instead of visual insect counting, which is extremely difficult and time-consuming [68,69]. The latter can also be assessed using mathematical modeling strategies based on population models [36,70] or through smartphone devices and machine vision [71], image analysis and machine learning [72], and deep learning [73]. However, the e-nose model with R = 0.99 was more adequate, accurate, practical, and is a low cost method. Even though ANN models were selected as best compared to SVM, the authors also have the latter models available for deployment depending on future usage needs.

**Figure 10:**
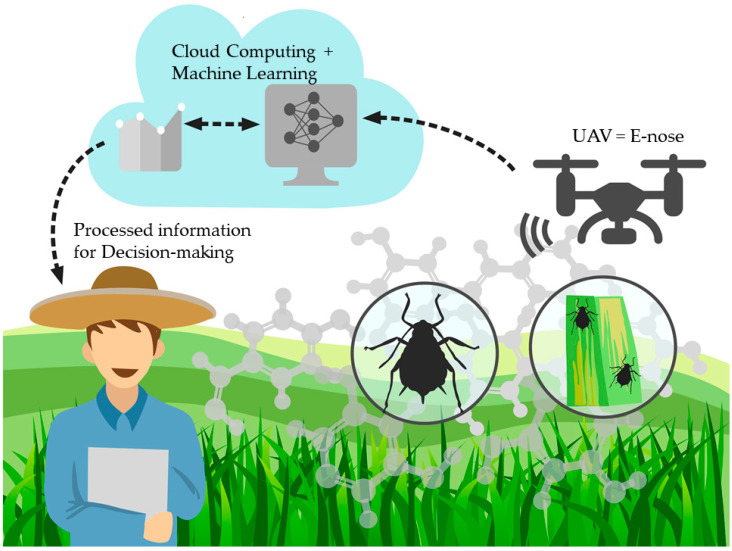
Diagram showing the proposed validation and deployment of machine learning models developed for early detection of aphids in wheat fields using an unmanned aerial vehicle and the e-nose as payload.

The deployment method for the e-nose proposed is as a payload for a UAV (Figure 10); the advantage of the e-nose is that it weighs only 200 g, and power can be accessed via the UAV. To assess the sensitivity and efficacy of the models, it is proposed to start flights at 5, 15, 20, and 50 m from the crop’s surface to test the ML models.

## 5. Conclusions

The low cost and accuracy of the models presented in this study could make the early detection of insect infestation in crop fields feasible using the UAV system proposed. The data and models used in this study can be used as a base for deployments in wheat fields and validation points considering other insects of interest. Further models developed following the phenological stages of plants can be used as testing systems for agronomical management practices for insect control, such as chemical and organic product applications, the introduction of natural predators, and integrated pest management tools. Furthermore, plant physiology models based on the low-cost e-nose opens the use of models to assess other biotic and abiotic stress effects on plants for further management practices such as fertilization, irrigation scheduling, and the general effect of climate change and climatic anomalies, such as heatwaves, frosts, and smoke contamination due to bushfires.

## Figures and Tables

**Table 1:** Results from the physiological data of the four treatments. Numbers on the top represent the mean values, while numbers at the bottom are the standard error.

Sample/ Parameter	Photosynthesis (µmol CO_2_ m^−2^ s^−1^)						Stomatal Conductance (mol H_2_O m^−2^ s^−1^)						Transpiration (mmol H_2_O m^−2^ s^−1^)					
**Measurement**	BL	D3	D7	D10	D14	D17	BL	D3	D7	D10	D14	D17	BL	D3	D7	D10	D14	D17
**Control**	6.78	9.18 ^a^	13.78 ^a^	12.47 ^a^	13.22 ^a^	12.75 ^a^	0.16	0.32 ^a^	0.51 ^a^	0.55 ^a^	0.50 ^a^	0.62 ^a^	2.35	3.60 ^a^	4.84 ^a^	4.16 ^a^	4.00 ^a^	6.00 ^a^
	±0.32	±0.01	±0.13	±0.30	±0.02	±0.12	±0.16	±0.01	±0.05	±0.25	±0.02	±0.09	±0.24	±0.02	±0.06	±0.28	±0.02	±0.06
**Low**	4.50	9.65 ^ab^	11.34 ^b^	10.49 ^b^	11.42 ^b^	10.27 ^b^	0.07	0.28 ^a^	0.35 ^c^	0.36 ^b^	0.37 ^bc^	0.40 ^c^	1.29	3.09 ^b^	3.81 ^c^	3.53 ^b^	2.95 ^c^	4.96 ^b^
	±0.23	±0.01	±0.09	±0.40	±0.02	±0.13	±0.41	±0.03	±0.16	±0.27	±0.02	±0.12	±0.27	±0.02	±0.10	±0.23	±0.02	±0.11
**Medium**	7.03	7.52 ^c^	12.19 ^b^	10.37 ^b^	10.70 ^b^	10.84 ^b^	0.15	0.16 ^b^	0.44 ^ab^	0.36 ^b^	0.33 ^c^	0.51 ^b^	2.19	2.11 ^c^	4.34 ^b^	3.12 ^b^	2.79 ^c^	5.36 ^b^
	±0.34	±02	±0.16	±0.65	±0.02	±0.20	±0.19	±0.02	±0.13	±0.33	±0.03	±0.17	±0.34	±0.03	±0.19	±0.24	±0.03	±0.15
**High**	7.03	10.93 ^a^	13.49 ^a^	10.67 ^b^	13.25 ^a^	11.07 ^b^	0.18	0.27 ^a^	0.44 ^b^	0.35 ^b^	0.44 ^ab^	0.50 ^b^	2.47	2.73 ^b^	4.43 ^b^	3.21 ^b^	3.53 ^b^	5.34 ^b^
	±0.37	±0.02	±0.15	±0.34	±0.01	±0.09	±0.22	±0.02	±0.07	±0.21	±0.02	±0.10	±0.25	±0.02	±0.09	±0.30	±0.02	±0.11

Abbreviations: BL: baseline; D: Day. Different letters denote significant differences between treatments according to ANOVA (*p* < 0.05) and Tukey honestly significant difference *post hoc* test (α = 0.05).

**Table 2:** Machine learning regression models based on artificial neural networks (Bayesian Regularization) to predict physiological data using the electronic nose outputs as inputs. Abbreviations: R: correlation coefficient; b: slope; MSE: means squared error.

Stage	Samples	Observations	R	b	Performance (MSE)
**Model 1—General (all treatments and measurement days)—10 neurons**					
**Training**	1008	3024	0.87	0.75	0.05
**Testing**	432	1296	0.83	0.75	0.06
**Overall**	1440	4320	0.86	0.75	-
**Model 2—Baseline and control—10 neurons**					
**Training**	378	1134	0.95	0.90	0.02
**Testing**	162	486	0.93	0.90	0.04
**Overall**	540	1620	0.94	0.90	-

**Table 3:** Machine learning pattern recognition models based on artificial neural networks (Levenberg–Marquardt) to classify samples into infestation treatment levels using the near-infrared absorbance values as inputs. Abbreviations: MSE: means squared error.

Stage	Samples	Accuracy	Error	Performance (MSE)
**Model 3—Baseline + Day 3—10 neurons**				
**Training**	404	100%	0.0%	<0.01
**Validation**	86	88.4%	11.6%	0.05
**Testing**	86	88.4%	11.6%	0.05
**Overall**	576	96.5%	3.5%	-
**Model 4—Day 7—10 neurons**				
**Training**	202	100%	0.0%	<0.01
**Validation**	43	95.3%	4.7%	0.02
**Testing**	43	93.0%	7.0%	0.02
**Overall**	288	98.3%	1.7%	-
**Model 5—Day 10—7 neurons**				
**Training**	202	100%	0.0%	<0.01
**Validation**	43	97.7%	2.3%	0.01
**Testing**	43	95.3%	4.7%	0.02
**Overall**	288	99.0%	1.0%	-
**Model 6—Day 14—10 neurons**				
**Training**	202	100%	0.0%	<0.01
**Validation**	43	90.7%	9.3%	0.05
**Testing**	43	86.0%	14.0%	0.04
**Overall**	288	96.5%	3.5%	-
**Model 7—** **Day 17—10 neurons**				
**Training**	202	100%	0.0%	<0.01
**Validation**	43	97.7%	2.3%	0.01
**Testing**	43	97.7%	2.3%	0.01
**Overall**	288	99.3%	0.7%	-

**Table 4:** Machine learning pattern recognition models based on artificial neural networks (Bayesian Regularization) to classify samples into infestation treatment levels using the electronic nose outputs as inputs. Abbreviations: MSE: means squared error.

Stage	Samples	Accuracy	Error	Performance (MSE)
**Model 8—Baseline + Day 3—3 neurons**				
**Training**	336	99.7%	0.3%	<0.01
**Testing**	144	95.1%	4.9%	0.02
**Overall**	480	98.3%	1.7%	-
**Model 9—Day 7—3 neurons**				
**Training**	168	100%	0.0%	<0.01
**Testing**	72	94.4%	5.6%	0.03
**Overall**	240	98.3%	1.7%	-
**Model 10—Day 10—3 neurons**				
**Training**	168	100%	0.0%	<0.01
**Testing**	72	97.2%	2.8%	0.01
**Overall**	240	99.2%	0.8%	-
**Model 11—Day 14—3 neurons**				
**Training**	168	98.8%	1.2%	<0.01
**Testing**	72	97.2%	2.8%	0.02
**Overall**	240	98.3%	1.7%	-
**Model 12** **—Day 17—3 neurons**				
**Training**	168	97.6%	2.4%	<0.01
**Testing**	72	86.1%	13.9%	0.06
**Overall**	240	94.2%	5.8%	-

**Table 5:** Machine learning regression models based on artificial neural networks (Bayesian Regularization) to predict the number of aphids’ data using the near-infrared absorbance values (Model 13) and electronic nose outputs (Model 14) from Days 7 to 17 as inputs. Abbreviations: R: correlation coefficient; b: slope; MSE: means squared error.

Stage	Samples	Observations	R	Slope	Performance (MSE)
**Model 13—NIR Day 7–Day 17—10 neurons**					
**Training**	605	605	0.99	0.97	555
**Testing**	259	259	0.94	0.98	3078
**Overall**	864	864	0.97	0.97	-
**Model 14—E-Nose Day 7–Day 17—10 neurons**					
**Training**	504	504	0.99	0.98	20,014
**Testing**	216	216	0.98	0.94	40,125
**Overall**	720	720	0.99	0.97	-

## Data Availability

Data and intellectual property belong to The University of Melbourne; any sharing needs to be evaluated and approved by the University.
